# Description of a new species of the genus *Ameletus* Eaton, 1885 (Ephemeroptera, Ameletidae) from Yunnan, China

**DOI:** 10.3897/zookeys.1021.59927

**Published:** 2021-03-01

**Authors:** Xianfu Li, Yanping Luo, Jian Jiang, Lili Wang, Xiaoli Tong

**Affiliations:** 1 Institute of Eastern-Himalaya Biodiversity Research, Dali University, Dali 671000, Yunnan, China Dali University Dali China; 2 Collaborative Innovation Center for Biodiversity and Conservation in the Three Parallel Rivers Region of China, Dali University, Dali, Yunnan, China South China Agricultural University Guangzhou China; 3 The Provincial Innovation Team of Biodiversity Conservation and Utility of the Three Parallel Rivers Region from Dali University, Dali, Yunnan, China Dali University Dali China; 4 Department of Entomology, College of Plant Protection, South China Agricultural University, Guangzhou 510642, Guangdong, China South China Agricultural University Guangzhou China

**Keywords:** COI, integrative taxonomy, Kimura 2-parameter, Mayfly, southwest China

## Abstract

A new species with primitive characteristics, *Ameletus daliensis* Tong, **sp. nov.**, is described, based on the morphology of imago, larva and egg with molecular data of the mitochondrial COI from Mount Cangshan, Dali, China. The new species is closely related to one of the most primitive mayflies, *Ameletus primitivus* Traver, 1939, by sharing persistent mouthparts in the alate stage, but it can be distinguished from the latter by the morphological differences of the mouthpart remains, wings and genitals in the imaginal stage. Both morphological and molecular evidence support that *A. daliensis* Tong, **sp. nov.** is a new member of the genus *Ameletus*. The discovery of the new species could help understand the origin and evolution of the genus *Ameletus*.

## Introduction

*Ameletus* Eaton, the largest genus of the family Ameletidae, is distributed in the Nearctic, Palearctic and Oriental Regions. The vast majority of the species of the genus are typical cold-water species and usually inhabit cold streams at mid-high latitude areas. Currently, *Ameletus* species are most diverse in the Nearctic Region with thirty-five species (Zloty 1996; Zloty and Pritchard 1997; Zloty and Harper 1999; Kondratieff and Meyer 2010). In the Palearctic Region, most species are distributed intensively in the East Palearctic areas, for example, about 12 species are known from the Russian Far East (Kluge 2007; Tiunova 2013; Tiunova et al. 2017), six species from Japan (Ishiwata 2001) and two species are reported from Korea (Bae and Yoon 1997). However, only four species are recorded from the Oriental Region (Traver 1939; Kang and Yang 2014). In China, *Ameletus* species have been paid little attention, with, so far, only five species being recorded: *A. costalis* (Matsumura) and *A. montanus* Imanishi are reported from north-eastern China (Quan et al. 2002); *A. atratus* Kang & Yang, *A. formosus* Kang & Yang and *A. montivagus* Kang & Yang are described from Taiwan, based on the larval stage (Kang and Yang 1994). During our recent survey on mayfly fauna of southwest China, many unknown species of *Ameletus* in the larval stage have been found, which suggests that the species diversity of *Ameletus* in the country is likely highly underestimated. Amongst them, an undescribed species was determined, based on larval and imaginal stages associated with laboratory rearing. Here, we describe this new *Ameletus* species by integrated approaches, including descriptions of imago, larva and egg and DNA sequence analysis (COI, Kimura 2-parameter).

## Materials and methods

**Figure 1.**
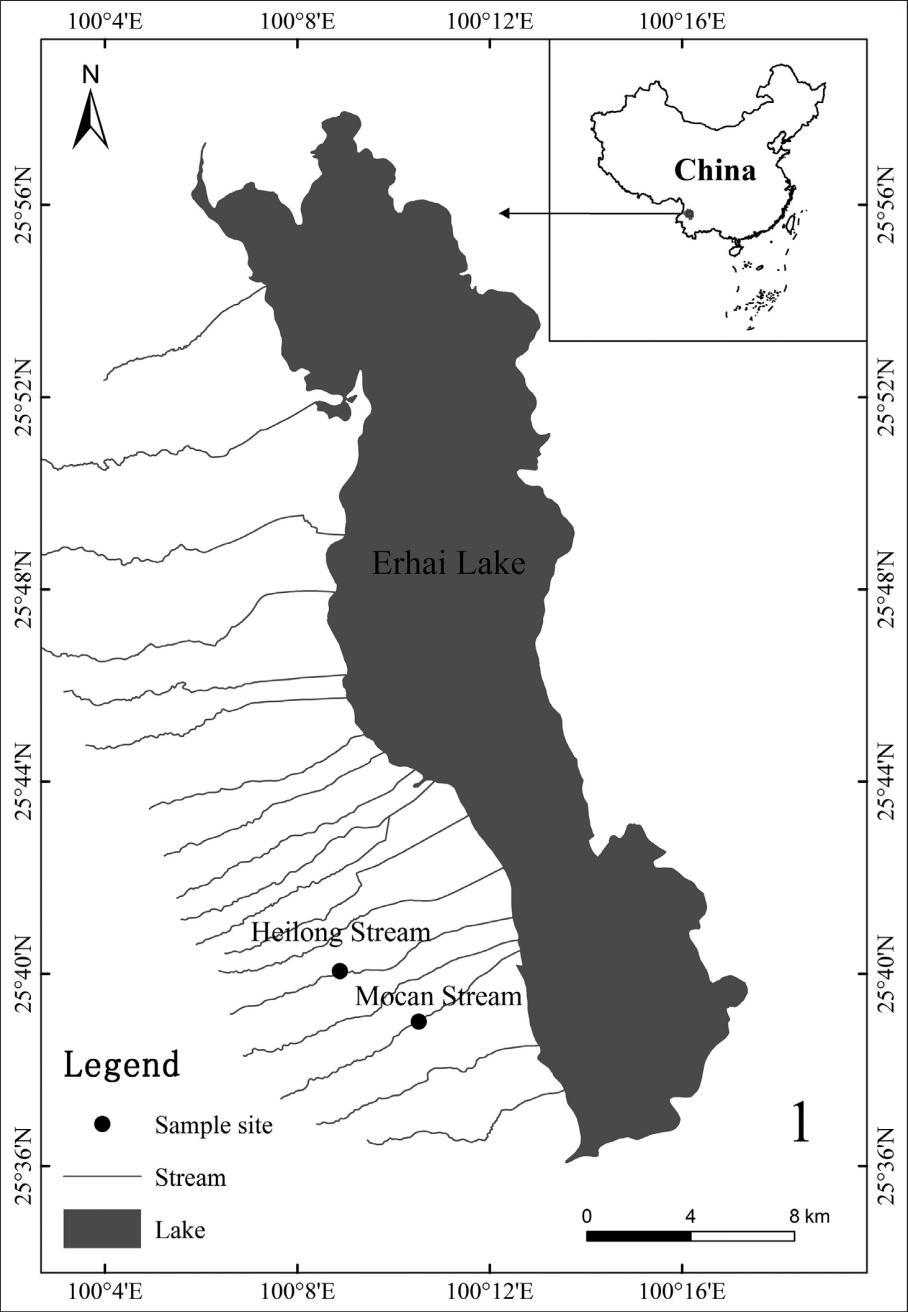
Map of collecting sites.

The specimens in this study were collected from Mount Cangshan, Dali City, Yunnan Province, China (Fig. 1). Mt. Cangshan, with 18 nearly parallel mountainous streams, is located at the southern end of the lofty Qinghai-Tibet Plateau and the southernmost mountain in Asia reached by the latest glaciation period. The summit of Mt. Cangshan reaches 4122 m a.s.l., the elevation of our collecting sites being between 2000 and 2250 m. The regional climate is influenced by plateau monsoons, being characterised by wet (May to October) and dry (November to April) seasons. The mean annual precipitation is 1683 mm, which is the major source of stream flow, while snow melt in the dry season is the minor source (Chiu et al. 2020).

The larvae were collected with a D-frame net from two small streams (Heilong and Mocan) in Mt. Cangshan, some of the larvae were then directly placed into vials containing 90% ethanol in the field, the mature larvae with black wing pads were selected for transportation to artificial rearing cages in situ (Figs 40, 41) and some larvae were taken back to the laboratory for rearing individually. Photographs were taken using a Canon EOS 5D Mark IV camera with MP-E 65 mm macro lens and a digital microscope (Keyence VHX-5000). Slide-mounted specimens were examined and photographed under the microscope with a digital camera attached. Some specimens were dissected under the stereomicroscope and were mounted on slides with Hoyer’s Solution for examination under the microscope. The map of the sampling sites (Fig. 1) is made in QGIS Standalone Installer Version 3.10 and the DEM data pixel is 30 m provided by Geospatial Data Cloud site, Computer Network Information Center, Chinese Academy of Sciences (http://www.gscloud.cn). The holotype (mature male larva) and two paratypes (male imago and female larva) are deposited in the Museum of Biology, Institute of Eastern-Himalaya Biodiversity Research, Dali University (**MBDU**); the remaining paratypes are deposited in the Insect Collection, South China Agricultural University (**SCAU**), Guangzhou, China.

Total genomic DNA was extracted from the legs of larva using the TIANamp Genomic DNA Kit (TIANGEN, Beijing, China) according to the manufacture’s protocol. The cytochrome c oxidase subunit I (COI) gene was amplified by the universal primers LCO1490-JJ/HCO2198-JJ to obtain a 658 bp fragment corresponding to the DNA barcoding region (Astrin and Stüeben 2008). Polymerase chain reaction (PCR) conditions were referred to Wesener (2015). The COI sequence was assembled by SeqMan (DNASTAR, Inc). Sequence alignments were performed by the Mafft (codon) algorithm, then optimised with MACSE (Katoh and Standley 2013; Ranwez et al. 2018; Zhang et al. 2020). The Kimura 2-Parameter distances between sequenced species were calculated by MEGA 7.0 with default setting (Kumar et al. 2016).

## Results

### Morphological taxonomy

#### Ameletus daliensis

Taxon classificationAnimaliaEphemeropteraAmeletidae

Tong sp. nov.

1264241E-A2BA-5B10-917F-639A1187BD60

http://zoobank.org/C2F34991-74B7-4842-8557-F97F6FEE5578

Figs 2–3 , 4–19 , 20–25 , 26–29 , 30–34 , 35–39

##### Material examined.

***Holotype:*** male mature larva (in ethanol, deposited in BMDU), **China**, Yunnan Province, Dali City, Mt. Cangshan, Mocan Stream (2020 m a.s.l.), 15.v.2020, coll. Xianfu Li. ***Paratypes*** (in ethanol, one male imago reared from larva and one larva are deposited in BMDU, the remaining in SCAU): 14 larvae and two imagos reared from larvae with same data as holotype; 20 larvae, one female sub-imago and one male imago reared from larvae, **Yunnan**, Dali City, Mt. Cangshan, Heilong Stream (2220 m a.s.l.), 1.v.2018, coll. Xianfu Li; one male sub-imago, Dali City, Mt. Cangshan, Heilong Stream, 28.v.2019, coll. Xianfu Li.

##### Diagnosis.

***Larva*** has the following combination of characters: 1) body with contrasting colour pattern; 2) labrum ventrally bordered with row of dense feathered setae (rare bi-forked setae) along anterior margin; 3) inner margin of trochanter in hind leg bearing row of brush-like fine and dense setae; 4) abdominal tergites I–X each with pointed spines on posterior margin; sternites without any spines on posterior margin, except V–VIII with tiny spines laterally; sternite IX with deep V-shaped cleft in both sexes. ***Sub-imago*.** 1) labial and maxillary palpi present and clearly visible; 2) wings semi-transparent, all cross-veins bordered around by dark brown. ***Imago*.** 1) labial and maxillary palpi present, but vestigial; 2) forewing transparent, MP2 turns downwards to meet CuA, stigmatic area suffused with milky and divided by a longitudinal vein; hind wings hyaline with short costal projection near the base; 3) genital forceps dark brown, apices of lobes round and slightly bent inwardly, ventral plates absent.

##### Description.

**Figures 2, 3.**
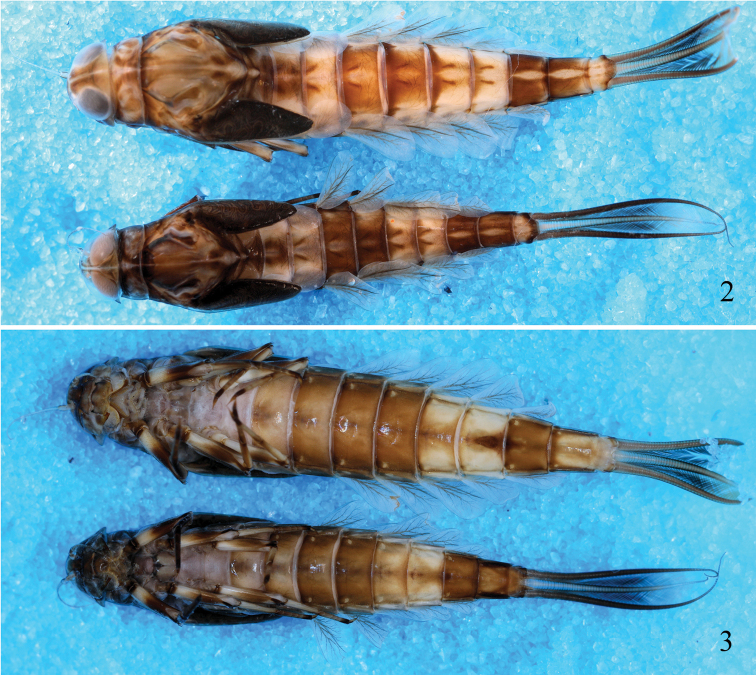
Larval habitus of *Ameletus daliensis* Tong, sp. nov. **2** dorsal view (upper: female; lower: male) **3** ventral view (upper: female; lower: male).

***Mature larva*** (in ethanol) (Figs 2, 3). Body length 14 (12.5–15.0) mm; cerci 7 (6.0–7.5) mm. Head brown, except ocelli pale. Eyes blackish-grey. Antenna brown dorsally at base, flagellum light brown. Clypeus brown; labrum mainly brown with two longitudinal dark brown stripes submedially. Pronotum dark brown with light brown irregular markings and one pale mesal line, meso- and metanotum brown with some irregular dark brown streaks and markings. Legs largely brown, except femora with pale patches on sub-basal and sub-distal areas, tarsi dark brown near apex. Abdominal tergites with contrasting colour pattern, tergite I white with diffuse light brown in a form of triangle medially, tergites II–III and VI–VII white, each with pair of diffuse light brown longitudinal bends sub-medially, tergites IV–V and VIII–IX mainly brown, each with longitudinal pale stripe medially, tergite X white with brown along posterior margin and pair of longitudinal light brown streaks; tergites II–IX each with pair of dark brown oblique stripes sub-medially and pair of dark brown stripes on sides; abdominal sternites brown, except sternites I, VI, VII and IX paler; sternites II–VIII each with ganglionic marking medially (Fig. 3), II–IX each with pair of small pale spots on anterolateral corners. Generally, the above colour pattern can change slightly in intensity, depending on the life stage.

**Figures 4–19.**
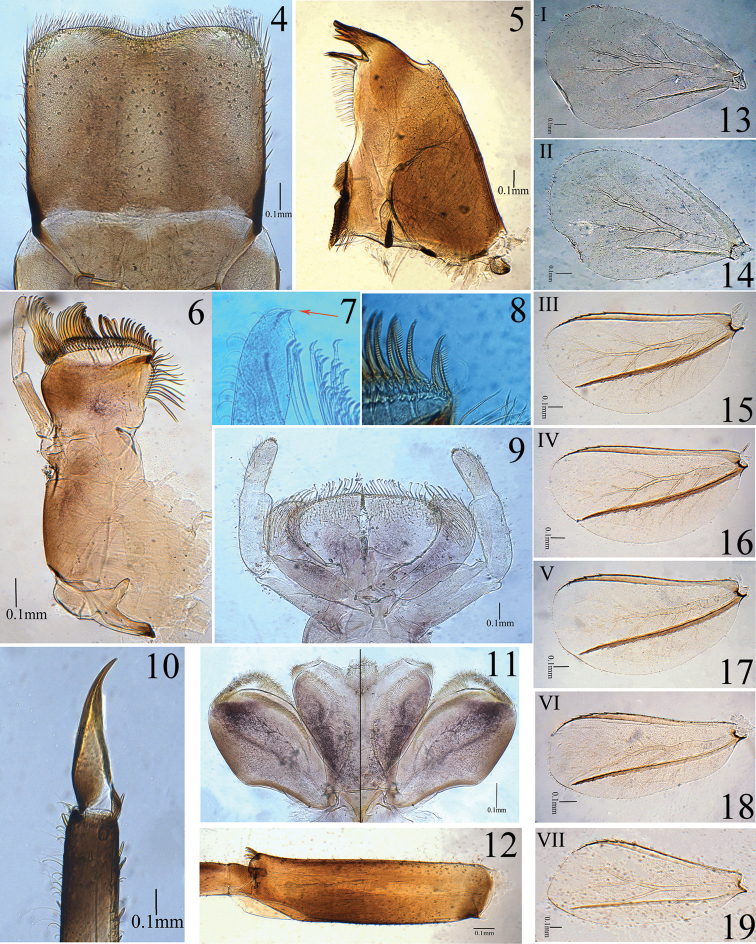
Larva of *Ameletus daliensis* Tong, sp. nov. **4** labrum (dorsal view) **5** right mandible **6** right maxillae **7** apex of terminal segment of maxillary palp (showing hook) **8** comb-shaped setae of maxilla (showing first seta) **9** labium **10** claw **11** hypopharynx (left: ventral view; right: dorsal view) **12** femur of foreleg **13–19** gills I–VII.

***Head*.** Flagellum of antenna with approximately 15–17 segments. Labrum (Fig. 4) rectangle (length to width ratio approximately 0.7:1) with shallow indentation on anterior margin, ventrally bordered with row of dense feathered setae (rarely bi-forked setae) along anterior margin. Outer incisor of left mandible with 4 denticles, first denticle longest, rest gradually getting shorter; outer incisor of right mandible with 3 denticles (Fig. 5), first denticle longest, second denticle slightly shorter or subequal to third. Hypopharynx as in Fig. 11, lingua with one median projection covered with hair-like fine setae. Right and left maxillae similar in structure (Fig. 6), crown of each maxilla with 27–31 comb-shaped setae and first seta with approximately 20 pointed denticles (Fig. 8), lateral galealacinia with row of approximately 14 long, feathered setae; maxillary palp 3-segmented, length ratio from basal to apical segments = 2.3:1.4:1, apex of terminal segment with one small hook (Fig. 7). Apical margin of glossae truncate and straight with row of long, spatulate flat setae widened towards apex (Fig. 9).

***Thorax*.** Dorsal surface of legs covered with many minute spine-like setae; apices of femora with crosswise row of distinct stout spine-like setae (Fig. 12): fore, middle and hind femora with 7–8, 4–5 and 3 stout setae at apices, respectively. Claws slightly curved and without denticles (Fig. 10). Inner margin of trochanter in hind leg bearing row of brush-like fine and dense setae (Fig. 23), fore and middle trochanters without such setae.

**Figures 20–25.**
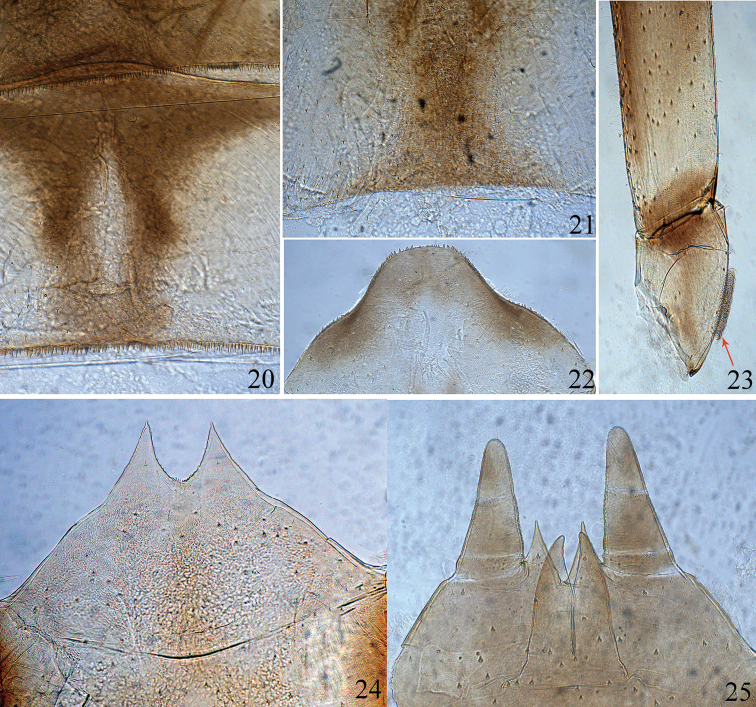
Larva of *Ameletus daliensis* Tong, sp. nov. **20** abdominal tergites VII–VIII **21** sternite VIII **22** abdominal tergite X (male) **23** trochanter of hind leg **24** female sternite IX **25** male sternite IX (showing penis buds, ventral view).

***Abdomen*.** Tergites I–X each with pointed spines on posterior margin (Figs 20, 22); sternites I–IV without any spines on posterior margin, V–VIII with tiny spines (Fig. 21) on posterior margin laterally (visible only under high magnification); surfaces of tergites and sternites I–IV without spine-like setae, but V–IX covered with tiny spine-like setae (Fig. 20), sternite IX with deep V-shaped cleft in both sexes, female with acute dentate emargination medially (Fig. 24), male without any denticles, penis buds without spine-like setae (Fig. 25); posterolateral spines on abdominal segments VIII–IX relatively short. Gills on abdominal segments I–VII (Figs 13–19); gills I–II white and oval, widest at apical half, each with short costal and anal ribs (Figs 13, 14); gills III–VII white with brown ribs and black tracheae, each with one strong costal rib and distinct serrations on costal margin and with one strong anal rib far from anal margin (Figs 15–19). Ratios of maximum width to length: gill I = 0.71, gill II = 0.66, gills III–IV = 0.48, gill V–VI = 0.50 and VII = 0.45. Cerci dark brown and median caudal filament paler (Figs 2, 3).

***Male imago*** (in alcohol). Length (mm): Body 13 (12.5‒14.0); forewings 12 (11.5‒12.5); cerci 19 (16.0‒22.0).

**Figures 26–29.**
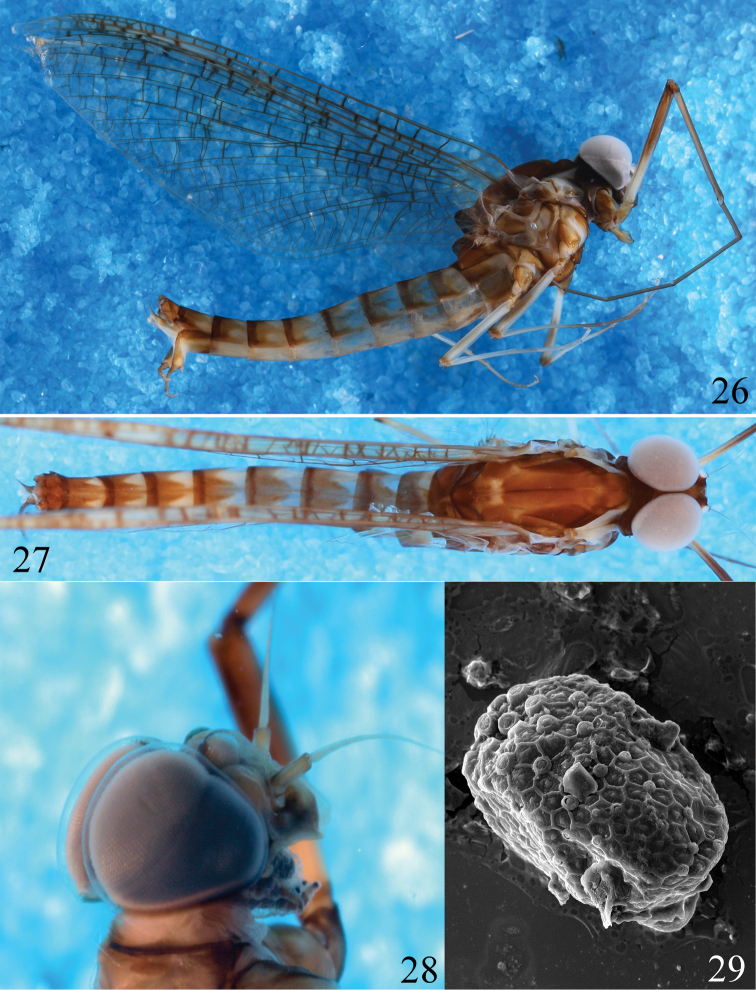
*Ameletus daliensis* Tong, sp. nov. **26** male imago **27** male imago (dorsal view) **28** head of male sub-imago **29** egg (SEM).

**Figures 30–34.**
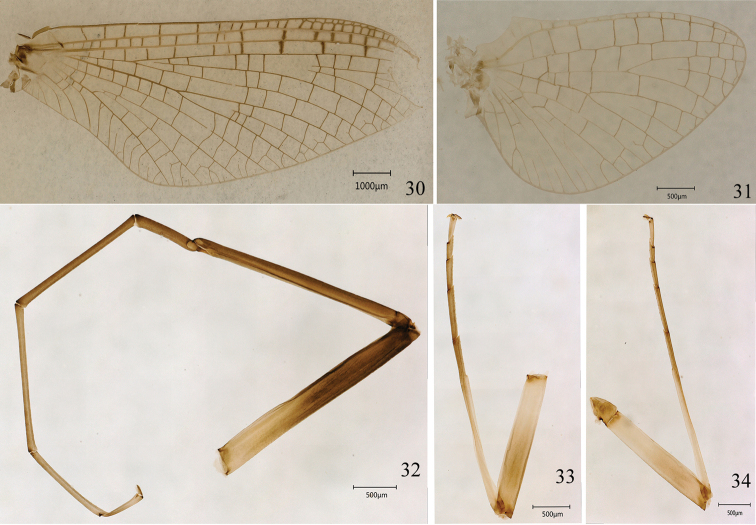
Imago of *Ameletus daliensis* Tong, sp. nov. **30** forewing **31** hind wing **32** fore leg **33** middle leg **34** hind leg.

***Head*.** Upper portion of compound eyes grey, lower portion dark grey (Figs 26, 27). Antennae light brown. Ocelli whitish. Labial and maxillary palpi present, but vestigial. ***Thorax***: Pronotum dark brown. Anteronotal protuberance brown, posterolateral sides dark brown; medioscutum brown, submedioscutum dark brown, median longitudinal suture dark brown (Fig. 27); sublateroscutum brown to dark brown; posterior scuttle protuberance brown with narrow white patch posterolaterally; scutellum brown, infrascutellum dark brown, scuto-scutellar impression light brown with pale lateral margins. Foreleg dark brown, except light yellowish-brown at basal 1/3 of femur (Fig. 32); middle and hind legs similar in colour and lighter than forelegs (Figs 33, 34); tibia pale and tarsus light brown, dorsal surface without spinules. Length of foreleg segments (mm): femur 2.7; tibia 2.7; tarsal segments 0.8, 1.7, 1.6, 1.1 and 0.5. Fore wings membrane transparent (Fig. 30), all veins dark brown with cross-veins lighter. Vein RS forked at about 1/4 of distance from base to margin; MA forked at middle of wing; MP_2_ turns downwards to meet CuA; stigmatic area suffused with milky and divided by longitudinal vein; cross-veins between C and R veins bordering around by dark brown. Hind wings hyaline with short costal projection near the base; vein MA forked at middle with one intercalary vein between MA_1_ and MA_2_; MP forked about one-third of distance from base to margin (Fig. 31).

***Abdomen:*** Tergites I and X brown, tergites II–IX brown with two triangle-like white markings on anterior half (Fig. 27). Sternites II–VIII pale, each with ganglionic marking medially. Cerci dark brown.

**Figures 35–39.**
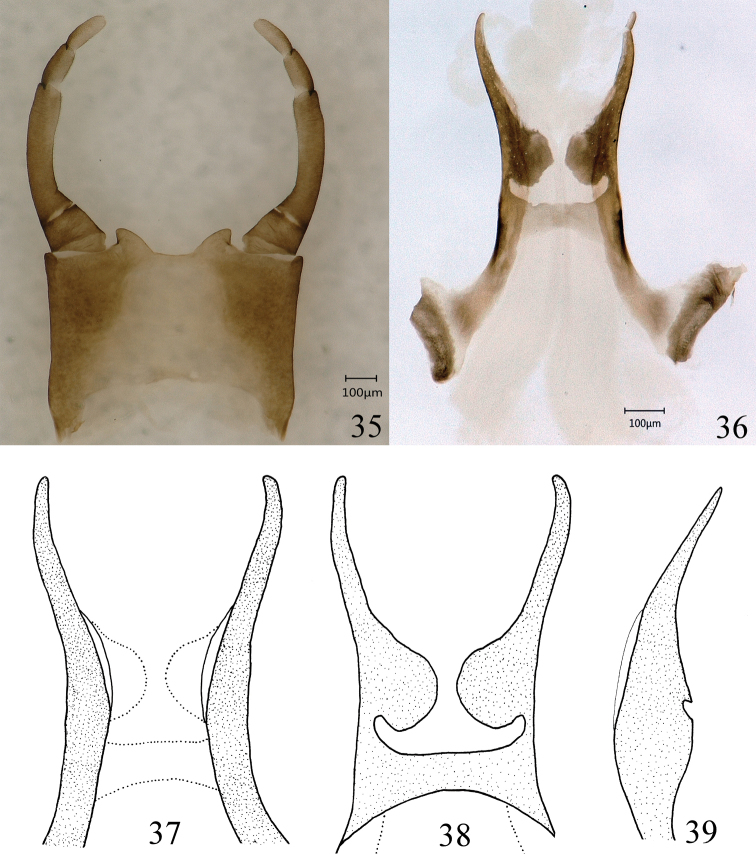
Male genitals of *Ameletus daliensis* Tong, sp. nov. **35** terminal abdominal sternites **36** penis lobes **37** penis lobes (ventral view) **38** penis lobes (dorsal view) **39** penis lobes (lateral view).

***Genitals:*** Styliger white with brown markings laterally (Fig. 35); forceps dark brown, terminal segment paler (Fig. 35); penis lateral lobes with spinules, apices of lobes round and slightly bent inwardly; ventral plates absent (Figs 36–39).

***Female subimago*** (in alcohol). Length (mm): Body 13.5; forewings 13.5; cerci 11. Compound eyes dark grey. Lateral view of head, the labial and maxillary palpi present and clearly visible (Fig. 28). Pronotum pale. Medioscutum and median longitudinal suture pale to light brown; submedioscutum and sublateroscutum brown. Wings semi-transparent, all cross-veins bordered around by dark brown. Abdominal tergite I pale with brown markings laterally and medially, colour pattern of other tergites similar to those of male; sternites II–VIII pale, each with blackish ganglionic marking medially, subgenital plate brown with deep V-shaped cleft.

***Eggs*.** Generally long ellipsoid shape with length 180‒205 μm and width 100‒115 μm (Fig. 29). The chorionic surface is covered by large-mesh polygonal cells, each cell with a small protuberance in the middle; large prominent round knobs exist on one pole only.

##### Etymology.

The specific epithet is named after the type locality, Dali City, Yunnan Province, China.

##### Distribution.

China (Yunnan).

##### Biology.

Larvae of this new species prefer to live in pools or slow currents with boulder and cobble substrate in very clear small streams. At one representative location (Heilong Stream, Mt. Cangshan) in May, the average water temperature was 14.6 °C, pH was 6.9 and DO (mg/l) was 7.6. Mature larvae with black wing-pads could be collected from early May through to mid-July, which suggests that the emergence period of the alate stage occurs from early May to late July, from which we infer that *A. daliensis* Tong, sp. nov. is a univoltine species in Dali, Yunnan. Before emergence, the mature larvae crawled to stones protruding from the water, half submerged and moulted to sub-imago (Fig. 42). The sub-imagos usually emerged on a warm sunny daytime and were rarely collected by light-trap in the evening.

**Figures 40–42.**
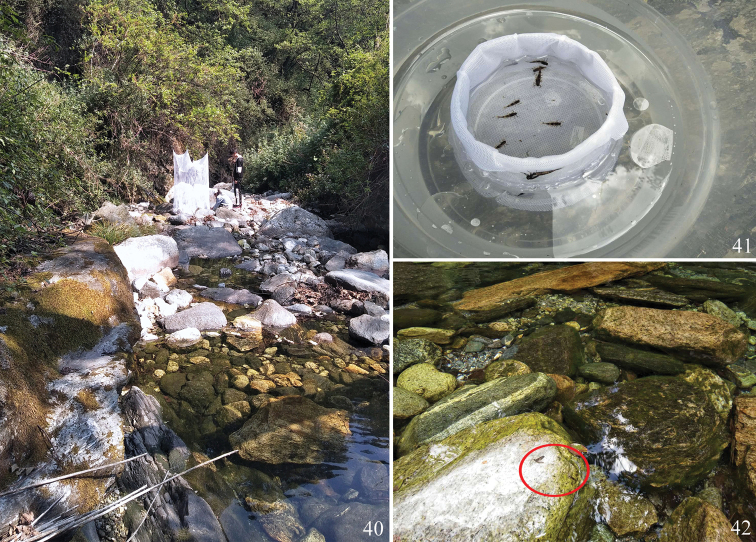
**40** rearing cage in the field **41** rearing cage **42** habitat of *Ameletus daliensis* Tong, sp. nov.

### Molecular analyses

A total of 71 COI sequences of *Ameletus*, which represent all sequenced species of the genus, including 24 unidentified species and 33 valid species, with four sequences from *Ametropus neavei*, *Baetisca lacustris*, *Metreletus balcanicus* and *Siphlonurus quebcensis* used as outgroups, were obtained from GenBank and BOLD for calculating genetic distances by K2P. The results showed that the interspecific genetic distances between *A. daliensis* sp. nov. and other sequenced *Ameletus* species are ranging from 5.5%–26.3%, of which the lowest K2P distance of 5.5% (Tables 1, 2) was found between the new species and *Ameletus* sp.1 MT-2014 (GenBank # KM207086 and KM244682, see Tang et al. 2014) from Sichuan, China. In general, 3.5% sequence divergence (K2P = 0.035) is considered as a likely maximal value for intraspecific divergence (Hebert et al. 2003; Ball et al. 2005; Zhou et al. 2010), although there is an exceptional case with 6% intraspecific distance in a mayfly in North America (Ball et al. 2005). The evidence of morphological differences in their larval stage and the genetic distance higher than 3.5% support the erection of the new species *A. daliensis* sp. nov.

**Table 1.** Collection information of the sequenced specimens from China.

Species	Collection locality	Collection date	GenBank Accession	Sources
*Ameletus daliensis* sp. nov.	Yunnan	15 May 2020	MW147549	This study
*Ameletus* sp.1 MT-2014	Sichuan	28 May 2012	KM207086.1	Tang et al. (2014)
*Ameletus* sp.1 MT-2014	Sichuan	28 May 2012	KM244682.1	Tang et al. (2014)

**Table 2.** Pairwise genetic distances (COI) between *Ameletus daliensis* Tong, sp. nov. and *Ameletus* sp.1 MT-2014 using the Kimura 2-parameter.

Taxa		K2P genetic distances	
		1	2
1	*Ameletus daliensis* sp. nov.		
2	KM207086*Ameletus* sp.1 MT-2014	0.055	
3	KM244682*Ameletus* sp.1 MT-2014	0.055	0.00

## Discussion

The larvae of the new species are similar to *Ameletus formosus* Kang & Yang from Taiwan with a V-shaped cleft on the posterior margin of abdominal sternite IX, but it differs from the latter by: (1) anterior margin of labrum having a row of dense feathered setae (rarely bi-forked setae); (2) gills I–II much wider (width/length ratio is 0.66–0.71) than those of *A. formosus* and each gill bearing a short costal rib and (3) V-shaped cleft on sternite IX is much deeper and more acute than that of *A. formosus*. In addition, the trochanter of the hind leg of the new species in larvae bears a row of brush-like fine and dense setae (Fig. 23). We are not sure whether this feature is unique for new species or shared by all *Ameletus* species, as it has never been mentioned in literature. The imaginal genital of the new species is characterised by being devoid of ventral plates. Interestingly, this character is also shared with *A. inopinatus* Eaton from Palearctic, *A. primitivus* Traver from Oriental and *A. velox* Dodds from Nearctic Regions. However, the lateral lobes of the penis are bent into an S-shape in *A. inopinatus* and are curved mesally in *A. velox* (Zloty 1996; Kluge 2007), in contrast to *A. primitivus*, the lateral lobes of the new species being relatively shorter and wider. Compared to the imago and larva of other *Ameletus* species, *Ameletus daliensis* Tong, sp. nov. is most closely related to *A. primitivus* Traver by its sharing retaining maxillary and labial palpi in the alate stage, in particular, these palpi (even the segmentation) were clearly visible in the sub-imago (Fig. 28). *A. primitivus* was firstly described by Traver (1939), based on the female imago, female sub-imago and the larvae from northern India. Zloty (2001) re-described the male imago of *A. primitivus* Traver and discussed its relationship with other *Ameletus* species. As the persistent mouthparts are a unique character in the winged stage of Ephemeroptera, *A. primitivus* Traver was considered as one of the most primitive in all mayflies (Traver 1939; Zloty 2001). However, the imago of the new species can be readily distinguished from that of *A. primitivus* Traver by the following combination of characteristics: (1) although maxillary and labial palpi are externally visible, they are reduced in size and atrophied and lacking in chitinisation (unlike *A. primitivus*, which are elongated and easily visible in the imago, cf. Zloty 2001: fig. 1F); (2) all wings are transparent (in contrast to both wings stained with dark brown at the basal half in *A. primitivus*) and (3) lateral lobes of penis relatively shorter (length ratio of lateral lobes to the width of penis is about 1:1, while the ratio is about 2:1 in *A. primitivus*). The larvae of the new species could be separated those of *A. primitivus* by (1) costal rib of gills III–VII with serrations and lacking spine-like setae; (2) abdominal sternites lacking spines on posterior margin, except V–VIII with tiny spines laterally and (3) surfaces of abdominal segments I–IV lacking spine-like setae on surfaces. Obviously, the new species has the characteristics that fall somewhere between Oriental and Holarctic species. The discovery of this new species bridges the gap between *A. primitivus* Traver and other Holarctic *Ameletus* species and could help reveal the origin and evolution of the genus *Ameletus*.

## Supplementary Material

XML Treatment for Ameletus daliensis
